# Retraction: Amino acid synthesis loss in parasitoid wasps and other hymenopterans

**DOI:** 10.7554/eLife.65123

**Published:** 2020-12-01

**Authors:** Xinhai Ye, Shijiao Xiong, Ziwen Teng, Yi Yang, Jiale Wang, Kaili Yu, Huizi Wu, Yang Mei, Zhichao Yan, Sammy Cheng, Chuanlin Yin, Fang Wang, Hongwei Yao, Qi Fang, Qisheng Song, John H Werren, Gongyin Ye, Fei Li

Ye X, Xiong S, Teng Z, Yang Y, Wang J, Yu K, Wu H, Mei Y, Yan Z., Cheng S, Yin C, Wang F, Yao H, Fang Q, Song Q, Werren J, Ye G, Li F. 2020. Amino acid synthesis loss in parasitoid wasps and other hymenopterans. *eLife* **9**:e59795. doi: 10.7554/eLife.59795.

Published 19, October 2020

We are retracting the above *eLife* paper due to errors that have resulted in incorrect conclusions concerning amino acid pathway loss in hymenopterans and other insects. Three colleagues (J McCutcheon, A Wilson, and N Moran) together alerted us that our conclusions were inconsistent with other studies showing the same amino acid pathway losses early in metazoan evolution. Upon examination, we found that the problem arose because of our incorrect homology assignments of certain amino acid pathway genes to nonhomologous insect genes, resulting in inaccurate characterization of gene loss patterns. As a consequence, conclusions concerning amino acid pathway loss are incorrect, requiring the paper’s retraction. All authors have agreed to the retraction.

